# Ethical considerations about artificial intelligence for prognostication in intensive care

**DOI:** 10.1186/s40635-019-0286-6

**Published:** 2019-12-10

**Authors:** Michael Beil, Ingo Proft, Daniel van Heerden, Sigal Sviri, Peter Vernon van Heerden

**Affiliations:** 1 Institute of Health Sciences at PTHV, Pallottistr. 3, 56179 Vallendar, Germany; 2 Institute of Ethics at PTHV, Pallottistr. 3, 56179 Vallendar, Germany; 3 Melbourne, Australia; 4 Hadassah - Hebrew University Medical Center, POB 12000, 9112001 Jerusalem, Israel

**Keywords:** Artificial intelligence, Machine learning, Intensive care, Medical ethics, Prognostication

## Abstract

**Background:**

Prognosticating the course of diseases to inform decision-making is a key component of intensive care medicine. For several applications in medicine, new methods from the field of artificial intelligence (AI) and machine learning have already outperformed conventional prediction models. Due to their technical characteristics, these methods will present new ethical challenges to the intensivist.

**Results:**

In addition to the standards of data stewardship in medicine, the selection of datasets and algorithms to create AI prognostication models must involve extensive scrutiny to avoid biases and, consequently, injustice against individuals or groups of patients. Assessment of these models for compliance with the ethical principles of beneficence and non-maleficence should also include quantification of predictive uncertainty. Respect for patients’ autonomy during decision-making requires transparency of the data processing by AI models to explain the predictions derived from these models. Moreover, a system of continuous oversight can help to maintain public trust in this technology. Based on these considerations as well as recent guidelines, we propose a pathway to an ethical implementation of AI-based prognostication. It includes a checklist for new AI models that deals with medical and technical topics as well as patient- and system-centered issues.

**Conclusion:**

AI models for prognostication will become valuable tools in intensive care. However, they require technical refinement and a careful implementation according to the standards of medical ethics.

## Background

Prognosticating the course of critical illnesses and predicting the impact of interventions are major pillars of decision-making in intensive care [1, 2]. This involves models which describe the underlying disorders in reductionist ways. Simple mechanistic types of models are based on causal relationships, e.g., hypotension in a dehydrated patient will respond to fluid resuscitation. A substantial number of individuals in intensive care, however, present with more complex disorders [3]. The critical condition in these patients results from various processes evolving at different time scales and interacting in non-linear and stochastic ways. Due to the large number of parameters, predictions based on mechanistic modeling become impractical. The intensivist then has to apply statistical techniques comparing the most salient traits of an individual case with reference classes, i.e., homogeneous cohorts of patients with the same dominant disorder(s). Due to the necessary reduction and, thereby, selection of the patient’s features to compare, these models match the characteristics of individual cases with only variable accuracy and prognostication becomes imprecise [4].

New prognostication techniques from the field of artificial intelligence (AI) and machine learning can process large amounts of data and have already demonstrated promising results in clinical studies [5–7]. For example, mortality prediction in cohorts of patients after cardiac arrest was substantially better with an area under the receiver operating curve (AUROC) of 0.87 in comparison to conventional prediction scores with an AUROC of 0.8 [8]. This difference was reproduced by predicting mortality in intensive care patients with the AUROC for the new techniques being 0.88 and that for conventional scores 0.78 or less [9]. Regarding these developments, it appears to be irresponsible not to consider making these new techniques part of everyday practice [10]. The efficient use and fast proliferation of AI applications in other parts of society, fed by advances of machine learning technologies and autonomous decision-making, underline the acuity of this topic [11]. In medicine, however, there are no AI systems for prognostication in routine use so far. In contrast to classifying existing data, such as retinal images [12], prognostication of future events is complicated by interferences which are still to happen. This makes predictions especially sensible to environmental conditions and adds an additional layer of uncertainty. There are also concerns about the biases and robustness of new AI techniques caused by the principal design of these technologies, i.e., their dependency on the properties of data samples used for machine learning. These issues lead to a number of ethical problems for prognostication in clinical practice. This paper will discuss these problems as well as their impact on intensive care medicine.

## AI and machine learning

AI refers to computer-based techniques making decisions which require human-like reasoning about observed data [13]. Historically, AI technologies were based on explicit rules and logic trying to simulate what was perceived to be the thought processes of human experts [6]. However, many prognostic questions in medicine are “black box” problems with an unknown number of interacting processes and parameters. Applying a restricted number of rules does not match the complexity of these cases and, therefore, cannot provide a sufficiently accurate prognostication. New types of AI are based on machine learning and better suited to this task [14]. They include artificial neural networks (ANNs), random forest techniques, and support vector machines.

The main aspect of machine learning is that the specific parameters for an underlying model architecture, such as synaptic weights in ANNs, are not determined interactively by humans. Instead, they are learned using general-purpose algorithms to obtain a desired output in response to specific input data [15]. The structural characteristics of a particular method, such as the layered architecture of an ANN, together with the associated set of fitted parameters constitutes a model that can be used for making predictions for new data inputs [16]. Adapting the architecture of a particular AI technology to specific types of input data can enhance predictive performance. For example, “recurrent” ANNs, such as long short-term memory, are constructed in a way to improve sequential data processing to capture temporal dependencies [17]. Of note, there has not yet been an algorithm developed to determine which type and architecture of the AI model would be optimal for a specific task [18].

For each particular model architecture, there are many different and equally good ways of learning from the same data sample. The machine learning algorithms usually do not recognize a single best combination of model parameters if such an optimum exists at all [6]. They can infer one or several plausible parameter sets to explain observed data presented during the learning phase [16]. This technology, therefore, is considered data-driven. It can detect emergent patterns in datasets, but not necessarily causal links [19].

### Datasets in machine learning

AI models for the purpose of prognostication are based on input of static or dynamic data, i.e., time series, or a combination thereof. Accumulation of data over time to document trends can enhance prediction accuracy [5]. Of great importance is the processing of heterogeneous data from electronic health records [20]. Due to the dependency of machine learning on the properties of datasets for training, issues of data quality and stewardship are becoming crucial. In addition to the availability and usability, reliability of data is an important topic. It encompasses integrity, accuracy, consistency, completeness, and auditability of datasets [21].

Useful output data for prognostication are either numbers representing probabilities of future events, time intervals until these events, or future trajectories of clinical or functional parameters. In addition to predictions of death vs. survival, prognosticating quality of life trajectories is now becoming more important for guiding decision-making, notably in the elderly [22]. Although the general concept of quality of life is difficult to operationalize, there are readily observable markers, such as the ability to perform activities of daily living [23], frailty [24], or cognitive capacity [25], which may serve as surrogates.

### Specific problems of machine learning

Both the structural characteristics of the models as well as the machine learning process itself make these new AI technologies different from previous approaches to prognostication. The lack of explicit rules of how machine learning operates prevents an easy interpretation by humans. This problem is most pronounced in ANNs due to the multitude of non-linear interactions between network layers [6]. Moreover, some model types, notably ANNs, are known to produce unexpected results or errors from previously known input data with some, apparently irrelevant modifications that might be undetectable by human observers (“adversarial examples”). Whether a particular error is a one-off “bug” or evidence of a systemic failure might be impossible to decide with poorly interpretable machine learning methods [26]. This also makes generalization of AI models potentially dangerous and, therefore, is considered a major problem for many applications [27, 28].

The learning process itself can be compromised by over- and underfitting models to the specific characteristics of training data. Underfitting models already fail to account for the variability of training data. Overfitting leads to a good performance with training data, but can eventually harm the robustness of the model in future real world applications with some distributional shifts of input data, e.g., due to variable practice patterns in different countries [29].

## Ethical considerations

There are a growing number of recommendations and guidelines dealing with the issue of ethical AI [30]. The European Commission has recently published guidelines for ethical and trustworthy AI. According to these guidelines, special attention should be focused on situations involving vulnerable people and asymmetries of information or power. In addition to adhering to laws and regulations and being technically robust, AI must be grounded in fundamental rights, societal values, and the ethical principles of explicability, prevention of harm, fairness, and human autonomy [31]. These principles echo the prima facie principles of medical ethics—beneficence, non-maleficence, justice, and human autonomy [32]—which are aimed at protecting vulnerable patients in the context of uncertainty and social hierarchies.

### The principles of beneficence and non-maleficence

The concepts of human dignity and sanctity of life imply that the application of information technology in medicine must be beneficent and non-maleficent for the individual patient. However, the specific AI use case of prognostication has the potential to violate these principles. Communicating probabilities derived from cohort studies to individual patients carries the risk of false hope, false despair, or continuing uncertainty. A falsely optimistic prognosis based on, for example, an unsuitable dataset for training an AI model could trigger futile, i.e., potentially inappropriate interventions. A falsely pessimistic prognosis could become a self-fulfilling prophecy when left unchecked [33]. The problems of accuracy and uncertainty apply to all probabilistic methods for prognostication and not only to AI. A way to solve this dilemma is to personalize probabilities as much as possible, e.g., by taking into account more features describing the individual circumstances of patients. The use of new AI techniques, such as recurrent ANNs, to extract prognostic information from longitudinal datasets [34] may serve that purpose. This approach is of particular interest, since most patients in intensive care are not in a steady state. Thus, the individual time course of a condition could be more informative and its analysis more predictive and, hence, more beneficial for the individual patient, than the analysis of data from a singular point in time.

### The principle of justice

The principle of justice deals with the distribution of resources within a society and non-discrimination of individuals. Non-intentional injustice to individuals has become an important issue for AI. Ranking algorithms are of particular concern in many fields of application. Due to the data-driven nature of AI techniques, the selection of datasets for training is a major source of discrimination. The following scenarios are of particular importance in this regard:

- Cultural biases can be inadvertently propagated from different communities by overlooking implicit rules ingrained in the social or professional framework of a specific environment [10]. This problem has already been recognized for conventional prognostication scores [35] which have then been modified to incorporate environmental characteristics [36].
- Algorithms may assign a low chance of survival to previously disadvantaged patient groups whose social status had correlated with a discriminatory biomarker, e.g., body weight. Suitable strategies for testing models and continuously auditing results will reduce that risk [31].
- The historical definition of empirical disease categories pushes the specific condition in an individual into a potentially inappropriate frame to guide further management. New research based on unsupervised learning methods has identified new and prognostically meaningful disease phenotypes that fit individual cases more accurately [37, 38]. For example, Seymour et al. [37] identified four novel phenotypes of sepsis which differ with respect to biomarkers and mortality. By using this new stratification, patients with sepsis could eventually receive treatment that is more adaptive in timing and intensity.

A possible approach to prevent already recognized biases is to exclude certain parameters, such as age or gender, from the training of AI models. Importantly, this is a conscientious decision within the society that introduces new biases and might also be associated with a price to pay, such as a substantial reduction of model performance and, therefore, its usability. Conflicts may occur between the different levels of justice (societal vs. individual) and could eventually violate the respect for patients’ autonomy (see below).

### The principle of patients’ autonomy

The respect for patients’ autonomy acknowledges the capacity of individuals for self-determination and the right to make choices based on his/her own values and beliefs [39]. It is regarded by many ethicists as first among equals. Not surprisingly, some authors, however, consider that statement problematic, especially in relationship to the principle of (distributive) justice [32]. The respect of patients’ or their surrogates’ deliberate choices also encompasses dealing with seemingly irrational decisions. The spectrum ranges from refusal of life saving treatment in a recently healthy individual to demands for interventions in a patient dying from an incurable disease. This opens the debate for an ethical analysis of personal behavior [40]. Guidelines for responding to requests for potentially inappropriate (futile) therapies in intensive care emphasize the importance of this issue [41]. It is important to note that predicting the course of a critical condition in individuals remains uncertain on principle. The fundamental problem of falsifying individual beliefs in future events cannot be solved by new AI technologies.

An autonomous decision by the patient or a surrogate decision-maker requires a sufficient understanding of the relevant medical information as well as of the decision-making process within the medical community, such as adherence to guidelines. The latter condition enables a dynamic dialog, i.e., shared decision-making, during the often unpredictable course of critical diseases. However, it is unrealistic to assume that these conditions can be fulfilled in every case. Hence, the trust between patients, surrogates, and physicians still is a major pillar of decision-making in intensive care. Traditionally, the burden of ethical decision-making is put onto the medical staff who must guarantee that the patient or his/her surrogate is able to make a decision to the best of his/her capacity. By being the gatekeeper for information, the medical professional—regardless if that means human or any future implementation of AI—acts as the guardian of the patient’s autonomy. Moreover, it is crucial to take the belief system and expectations of the patient or his/her surrogate into account [42] to prevent a return to the paternalistic medicine of the past. That type of medicine was mostly based on calculations of non-maleficence and beneficence by physicians. Empirical studies, however, indicated that the trust of patients in the prognostication accuracy of physicians is rather low [43]. This finding is especially important when discussing irreversible decisions, such as withdrawing treatment after ranking quality of life higher than extending life at any cost. Patient-centered outcomes rarely are binary and can involve a broad range of expectations related to the self-perceived quality of life. Regarding AI models for prognostication, this fact requires more consideration for both the training samples for machine learning as well as for defining the types of output.

A difficult problem for shared decision-making is the discussion about probabilities while prognosticating future events. Regarding the uncertainty inherent in every prediction, probabilistic models are likely the best available instruments now. However, if one has to decide upon an individual case, i.e., sample size of 1, probabilities are meaningless irrespective of the mathematical validity of the model [4]. Although human intuition may indicate otherwise [44], there is no objective difference between a predicted mortality of 10 and 90% for the assessment of an individual case. Thus, any dispute about how to act on probabilistic information for an individual, especially on when and how to invest resources, cannot be resolved with data from AI models alone. Instead, there has to be a mutual agreement between the patient or surrogate and the physician about the interpretation of probabilities. A disagreement has the potential of interfering with the principles of autonomy and justice. A solution to that dilemma is an observation period to obtain longitudinal data for the individual patient and, thereby, reduce the uncertainty of prognostication [1].

### The principle of explicability

**Fig. 1:**
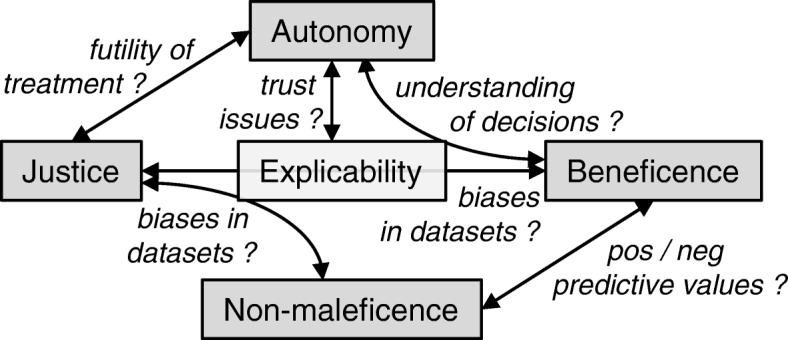
The interplay and potential areas of tensions between the four principles of medical ethics as well as with the principle of explicability

In addition to the four principles of medical ethics, recent guidelines for an ethical AI also dealt with the issue of explicability, i.e., transparency of models in producing outputs based on specific inputs [13, 31]. The interplay and potential tensions between these five principles is depicted in Fig. 1. Of note, the absence of insight into the mechanisms of data processing by AI models is not fundamentally different from the opacity of human thinking [10]. Without critical reflection, algorithmic tools, such as conventional prognostication scores, are handled by intensivists like “black boxes” [45]. However, humans can be requested to reason and justify their conclusions if there is a lack of certainty or trust. In contrast, there has not been a design framework yet in place that creates AI systems supporting a similar relationship. Current research into illustrating the models’ decision-making process in more transparent ways aims at distilling ANNs into graphs for interpretative purposes, such as decision trees [46], defining decision boundaries [47], approximating model predictions locally with interpretable models [48], or analyzing the specific impact of individual parameters on predictions [49]. Full explicability may not always be possible and other measures to audit outputs need to be implemented to assure that the principles of medical ethics are respected [31]. Trust in the working of AI algorithms and the ability to interact with them would enhance the patients’ confidence that is required for shared decision-making. Moreover, model transparency—as far as this might be achieved—also helps to clarify questions of moral and legal accountability in case of mistakes [10].

The design and deployment of AI systems also evoked discussions about societal values and fundamental rights with explicability being the focus of much controversy. Some scientists recommend sacrificing the power of AI models in favor of explicability to foster social trust and prevent domination by unaccountable models or algorithms [10]. Of note, if machine learning is considered empiricism, this issue has already been picked up by Aristotle more than two millennia ago. Large parts of the evidence base in medicine resulted from empirical studies. However, empiricism in prognostication that informs irreversible decisions in intensive care requires rules and boundaries. A quantitative assessment of uncertainty plays a major role in this regard [19, 50].

### Privacy and confidentiality

Privacy and confidentiality are still important values to protect human dignity as a fundamental right in many countries. New guidelines for data processing, notably the general data protection regulation (GDPR) in Europe, also contain demands to communicate risks created by the processing of patients’ data including profiling and its consequences [26].

## A pathway to an ethical AI-based prognostication

The guidelines for a trustworthy AI by the European Commission [31] list several requirements to translate the above ethics principles into practice (Table 1). Some of these requirements are directly derived from ethical principles, such as transparency and explicability. Others are prerequisites for implementing these principles, such as the requirement for human agency to support patients’ autonomy.

**Table 1:** Requirements for the implementation of ethics principles for a trustworthy AI (based on [31])

Leading ethics principle	Requirement for implementation
Beneficence	Data stewardship, accountability
Non-maleficence	Technical robustness
Justice	Fairness, societal wellbeing
Autonomy	Human agency and oversight
Explicability	Transparency

### Technical issues

**Fig. 2:**
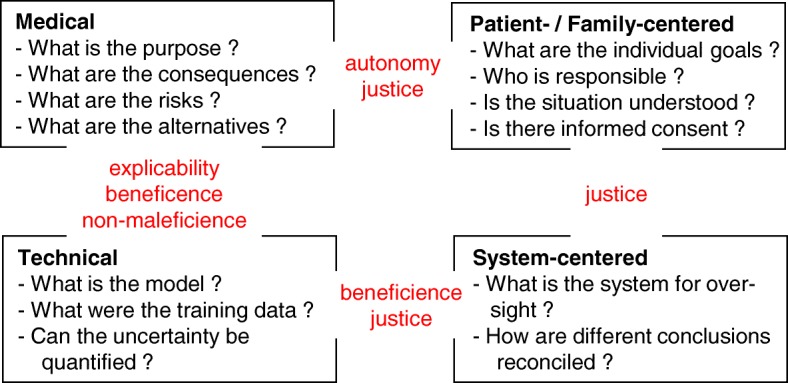
Classes of questions for evaluating AI-based prognostication models (the main ethical principles related to specific topics are depicted in red)

Based on these guidelines, we suggest a checklist to assess AI systems for the purpose of prognostication in intensive care (Fig. 2). This set of questions consists of four topics—medical, technical, patient-centered, and system-centered. After defining the purpose for prognostication, e.g., change of medical management, the selection of a suitable AI model is crucial. This concerns input and output data types, the architecture of the AI model, such as ANN or random forest, as well as the dataset used for training. The origin and composition of this dataset must be clearly defined to identify explicit and implicit biases. Ideally, the training dataset is from the same cultural background to minimize distributional shifts. Since no gold standard for AI model design has yet been elaborated, the process of model development might involve several iterations trialing various models to optimize performance. Human expertise and creativity might still be required for this task. Maximum explicability of the model should be sought by quantifying the role of individual parameters [49] and decision boundaries analysis [47]. This approach is not unlike the training of intensivists—the trainees initially possess a set of basic skills which they refine according to performance measures. While doing this, they have to explain their decisions including their reasoning.

The benefit of AI-based prognostication models is determined by their accuracy which can be described by the calibration at the cohort level and the quantification of discriminatory power and predictive uncertainty [29]. To adhere to the ethical principle of non-maleficence, model predictions cannot be actionable without confidence values for assessing uncertainty and, thereby, risks [19]. There is no standardized approach to estimate predictive uncertainty that results from the model architecture and the machine learning algorithm itself. However, recent research indicate that, for example, training multiple ANNs using random initialization and ensembling predictions could be a useful method to obtain quantitative estimates for uncertainty [29].

### Issues of decision-making

We think that shared decision-making with patients or their surrogates is the best way to finally decide upon the consequences of information from AI-based prognostication models. This approach also guarantees human oversight and, thereby, assigns responsibilities to the physician and the patient or surrogate. Assuming that the AI model was formally appropriate and tested, differences in predictions between that model and those by the medical staff should be dealt with by seeking additional (human) opinion, e.g., in multidisciplinary team meetings. This also concerns conflicts between patients or surrogates and medical staff in interpreting results [41].

### Societal issues

Governments have established systems for oversight to assure the competency of medical staff and, thereby, support trust in the health care system. New AI technologies should face a similar regulatory scrutiny that encompasses the design, configuration and operation of algorithms [26]. Moreover, medical staff should be trained to evaluate and supervise these systems as well as to discuss the characteristics of AI, including potential errors, with patients and surrogates. Finally, the general public should be educated in dealing with AI to better understand the merits and dangers of AI-based prognostication and become capable of joining the decision-making process when needed.

Table 2 summarizes the main steps of implementing AI models for prognostication as well as potential mistakes.

**Table 2:** Dos and don’ts while implementing AI-based prognostication models

Do	Don’t
Define individual goals	Rely on a single model
Clarify responsibilities	Use models without testing
Choose a suitable model	Make decisions in paternalistic ways
Assure technical robustness	Be cavalier with human oversight
Seek maximum transparency	Ignore data privacy issues
Quantify uncertainty	Underestimate the role of empiricism
Share decision-making	
Monitor performance and update model	
Contribute to research and education	

## Discussion and conclusions

Data-driven AI is considered a disruptive and transformative technology in medicine. It provides the opportunity to process a multitude of patient-level data to detect and specify disease patterns. Although powerful tools are being developed with this technology to predict event rates and risks at the population level, the prognostication of future events, disease trajectories, or functional outcome for the individual patient remains a fundamental problem. An important reason is the probabilistic nature of all prediction models derived from cohort data which are then applied for the individual case. Moreover, the stochastic nature of the interplay between the patient’s conditions and the environment adds a substantial amount of predictive uncertainty. Thus, individual prognostication is inherently uncertain. New information technologies can not eliminate but only reduce this uncertainty. For the sake of prognostication in the individual, it is less important to set an “acceptable” threshold for the accuracy of a particular model at the population level, but to create a framework to describe and handle uncertainty.

In anticipation of imperfect predictions for the individual and, consequently, potentially wrong decisions, rules are required to protect the dignity of patients as well as the integrity of medical professionals. These rules are ingrained in laws and guidelines as well as ethical standards with the latter being regarded superior to the others. The ability to make decisions under conditions of uncertainty and in compliance with the rules of medical ethics is considered a crucial competency of intensivists. AI-based techniques which, one day, might develop from assist systems into “artificial intensivists” will need to acquire the same competency. Whereas the accuracy of prognostication and, eventually, decision-making are evaluated in the setup of clinical trials, ethical problems have not yet received sufficient attention when discussing AI.

The main principles of medical ethics are beneficence, non-maleficence, justice, and respect for patient’s autonomy. Whereas compliance to beneficence and non-maleficence by AI models can be evaluated—at least partially—by standardized measures, such as sensitivity and specificity, compliance to the other two principles is more difficult to assess. Moreover, the latter two principles have the potential to be in conflict with each other and to turn individual interests against societal interests [32]. Importantly, the less well-understood mathematical characteristics of new AI technologies lead to a lack of transparency and problems with uncertainty quantification in the decision-making process. Both characteristics, however, are prerequisites to assess justice and implement the principle of patients’ autonomy through knowledge and understanding. Of note, a fully autonomous decision-making system could serve justice best at the level of society. However, such an approach would violate the principle of human autonomy and be ignorant of empathy that is considered a fundamentally human trait. These problems (cf. Fig. 1) are not restricted to medical applications and requires solutions in a wider context [51].

The principle of patient’s autonomy is widely seen as a major rule that governs the patient-physician relationship. It requires a comprehensive understanding of the medical issues to be dealt with by both sides. This is not realistic, especially when involving new technologies which are at the edge of today’s knowledge. Thus, the principle of patient’s autonomy has to be discussed in the context of trust in the competencies of medical professionals as well as in the system of oversight for new technologies. The concept of shared decision-making and the systems of legal responsibilities are based on this trust.

Currently, there are a large number of new guidelines on AI being issued by both professional and governmental institutions. These need to be synchronized and unified under universally accepted rules and limitations. This process will be iterative and consultative to take new developments into account. Moreover, educating the public as well as the members of the professions not traditionally involved in AI would create the foundation for a widespread acceptance of AI.

The implementation of new predictive AI technologies into the provision of intensive care medicine requires strict data governance measures which include safeguards for the integrity and quality of data. The governance of algorithms is also important. Many established prognostication methods can be considered “black boxes” when applied without in-depth knowledge. Thus, the status quo of prognostication cannot be regarded as a gold standard to evaluate new technologies. To guide a formal assessment of new prediction models, the performance of these models should be at least as good as that of experts or already established models as documented in clinical trials. This would allow for dynamic adjustments and ensure the quality of care at the systems level.

The current lack of explicability of many AI techniques is going to restrict their use to adjunct, e.g., decision-support, systems for a while. After conclusive evidence for their overall effectiveness and beneficence will become available, these new techniques will most likely turn into perceived standards. They will gain professional acceptance [52] and diverting from them will require justification [53]. Importantly, values in society evolve over time. Thus, continuous monitoring of AI model performance and patients’ outcome should become mandatory to calibrate these measures against ethical standards.

In conclusion, new AI and machine learning techniques have the potential to improve prognostication in intensive care. However, they require further refinement before they can be introduced into daily practice. This encompasses technical problems, such as uncertainty quantification, inclusion of more patient-centered outcome measures and important ethical issues notably regarding hidden biases as well as the transparency of data processing and the explainability of results. Thereafter, AI models may become a valuable component of the intensive care team.

## Data Availability

Not applicable.

## Abbreviations

AI: Artificial intelligence
AUROC: Area under the receiver operating curve
